# Development of a novel precision applicator for spot treatment of granular agrochemical in wild blueberry

**DOI:** 10.1038/s41598-024-64650-z

**Published:** 2024-06-14

**Authors:** Craig B. MacEachern, Travis J. Esau, Qamar U. Zaman, Scott N. White, Aitazaz A. Farooque

**Affiliations:** 1 Department of Engineering, Faculty of Agriculture, Dalhousie University, Truro, NS Canada; 2 Department of Plant, Food, and Environmental Sciences, Faculty of Agriculture, Dalhousie University, Truro, NS Canada; 3 Canadian Centre for Climate Change and Adaptation, University of Prince Edward Island, St Peters Bay, PE Canada

**Keywords:** Engineering, Materials for devices, Environmental impact

## Abstract

While spot spraying has gained increasing popularity in recent years, spot application of granule agrochemical has seen little development. Despite the potential for the technology, there currently exists no commercially available granular applicators capable of spot application. Therefore, the goal of this study was to design, build, and lab evaluate a precision applicator for spot applying granular agrochemical in wild blueberry. The design incorporated a John Deere RC2000 with a custom control box, recirculation system, and electrically actuated valves. All components were modified to fit a Valmar 1255 Twin-Roller. The system receives inputs from a predeveloped prescription map and can actuate each of the twelve valves separately to provide individual orifice control. Casoron® G4 was used as the testing agrochemical and in cycling the product pneumatically for 1 hour incurred no significant product degradation (*p* = 0.110). In lab evaluations, the applicator encountered zero errors in reading prescription maps and actuating the correct valves accordingly. Further, the granule recycling system had zero instances where product built up in the lines or jammed the valves. In all, this project represents the first successful development of a precision granular spot applicator for any cropping system.

## Introduction

Research into granular agrochemical application has largely centered around variable rate technologies. These technologies are typically prescription map based and allow the user to alter the application rate throughout a field in real time based on the map. Maps can be developed for a variety of purposes and based off a variety of parameters such as yield, slope, soil moisture, pest pressure or any other spatially referenced parameter^1^. Reference^2^ developed a variable rate fertilizer applicator that could adjust application rates detailed by a prescription map with accuracies ranging from 89.3 to 98.1% in terms of applied rate to target rate. Reference^3^ developed a similar variable rate applicator for use in rice. Again, their applicator was able to vary rates of fertilizer based on a prescription map with an accuracy ranging from 81.9 to 97.4%. Reference^4^ modified a granular applicator to accommodate variable rate application. Their system was able to maintain application rates within ± 5% of the target rate with a 0.95–1.90 s delay required to make any changes to the application rate. Reference^5^ developed a variable rate seed drill that maintained a coefficient of variation of 2.34% to 5.10% in terms of applied rate to target rate based on setup parameters. Reference^6^ developed a variable rate applicator for oil palm based on radio frequency identification (RFID). RFID tags were affixed to trees and fertilizer rates were adjusted automatically based on defined rates for each tree. The average inaccuracy achieved by their system was 0.74 m.

Today there are several commercially available applicators which can accommodate variable rate applications. Of the available options, Kuhn (Bucher Industries AG, Niederweningen, Switzerland) and Valmar (Linamar Corporation, Guelph, Canada) are among the larger, with offerings ranging in hopper capacity from 0.5 to 18.7 m^3^ and application widths from 7.3 to 50.0 m. Despite the plethora of commercial variable rate options for granular products, there has yet to be significant progress made toward a commercially viable granular spot applicator. Spot application would offer the unique potential to not only vary rate throughout a field, but the potential to turn on/off individual boom orifices to achieve a greater level of precision.

Spot application of granular products in field crops is largely unexplored in literature though Reference^7^, did develop a spot applicator for variable rate nitrogen in citrus groves which used prescription maps based on the ultrasonically sensed volume of tree canopies to vary fertilizer rates throughout the grove. Their system avoided application in areas without trees or areas with smaller trees which in effect is spot application. That said, the system designed by Reference^7^ is essentially an on/off for the entire application. The goal of a spot applicator in a field crop is to provide sectional control within the application extents meaning, individual orifices could be turned on/off across the boom individually, not simply cutting the entire application.

The basis for this work builds off the study done by Reference^8^, which explored the potential for spot application of granular fertilizer in wild blueberry. While their study demonstrated the significant potential of spot application of granules, their applicator design had several technical limitations which restricted its practical functionality. First, their decision system utilised pneumatic actuators to control product flow immediately after the metering wheel. This meant that when a positive detection was made, there was a delay of between 4.92 and 7.39 s before product was applied. The variability comes from a variety of factors including the length of hose the product must travel through, the air flow coming from the blower fan as well as the composition of the product being applied. For instance, the further the orifice is from the venturi, the farther the product must travel and the longer time that travel will take. This in theory can be calculated and accounted for however, that assumes that all other variables related to the flow remain constant. However, there are several reasons why this assumption is not reliable. First, the velocity of air flowing from the blower fan is slightly variable due to turbulence in the hosing. Second, when travelling in a straight line the delay between detection and application may be sufficient. However, when turning, the distance between the sensor and the orifice will change and the time it takes to travel to the detected point will likewise change. This will result in product being applied in incorrect areas or missing targets entirely if reliant on a static delay. This could potentially be mitigated by global navigation satellite system (GNSS) integration however this is an unnecessary complication if the delay can be avoided entirely. Finally, the composition of the applied material will likewise play a critical role in the delay timing. This was demonstrated in the results of Reference^8^ where delay time differences for a single orifice were as high as 1.31 s between clay filler and fertilizer. This equates to an on-ground difference in application point of 1.74 m at a ground speed of 4.8 km hr^-1^. While a bulk density component could be added to the calculation to mitigate this effect, it won’t solve the problem entirely and adds another variable in the calculation of the delay. For these reasons the major focus of this project was to mitigate or even remove the effect of the delay in the system.

The second major issue with the design used in Reference^8^ was the use of a screw auger to return unapplied product to the hopper. In testing the system, granules conveyed with the screw auger tended to get crushed between the cover and the screw itself. This resulted in significant product degradation and would result in changes to the application rate independent of any changes to the software inputs defining the application rate. As a further point of complication, by crushing the granules and altering the bulk density of the returned product, the delay timing of the system would likewise change. For these reasons, a design which did not alter the composition of the granules would be necessary in future iterations of the spot applicator.

The applicator produced in this work was designed to target hair fescue (*Festuca filiformis*) in wild blueberry fields. Hair fescue is a densely tufted perennial weed which has quickly become the pest of greatest concern for Nova Scotian wild blueberry producers^9^. The weed has rapidly spread since 2001 where it was only found in 7% of fields to being found in over 75% of fields by 2019^10^. The dense sods formed by hair fescue can reduce wild blueberry yields by over 50%^11^. Currently, pronamide is the only product which is widely employed for managing hair fescue in Canadian wild blueberry fields. Pronamide provides over 90% control of hair fescue^11,12^ though it does have some technical limitations in the form of its strict application window, temperature sensitivity, and cost^13,14^. Further, being that it is the only product with widespread implementation in hair fescue management, herbicide resistance mitigation needs to be considered. Dichlobenil, the a.i. in Casoron® G4 is the only registered alternative to pronamide which has demonstrated the ability to control hair fescue^15^. That said, its implementation remains limited due to its granular composition and cost of over CAD$1800 ha^-1^ for a broadcast application^14^. Despite this, Casoron® G4 has potential if a suitable means of cost reduction such as spot application can be realized. Considering hair fescue’s average field uniformity is only 37%^10^, there is a theoretical cost savings of 63% through spot application.

Given (1) The rapid spread and economically destructive nature of hair fescue in wild blueberry fields, (2) the wild blueberry industry’s overreliance on pronamide and the potential selection for herbicide resistance, (3) Casoron® G4’s significant potential for managing hair fescue, (4) Casoron® G4’s uniform application cost of $1873.19 ha^-1^ and (5) the lack of commercially available spot applicators for granular agrochemical; the objective of this study was to develop and lab evaluate a precision applicator for spot applying Casoron® G4 in wild blueberry. Such a development would serve to significantly reduce application costs and could be a viable solution for spot applying alternative granular agrochemicals in a variety of cropping systems.

## Methods

### Granular degradation trials

**Figure 1:**
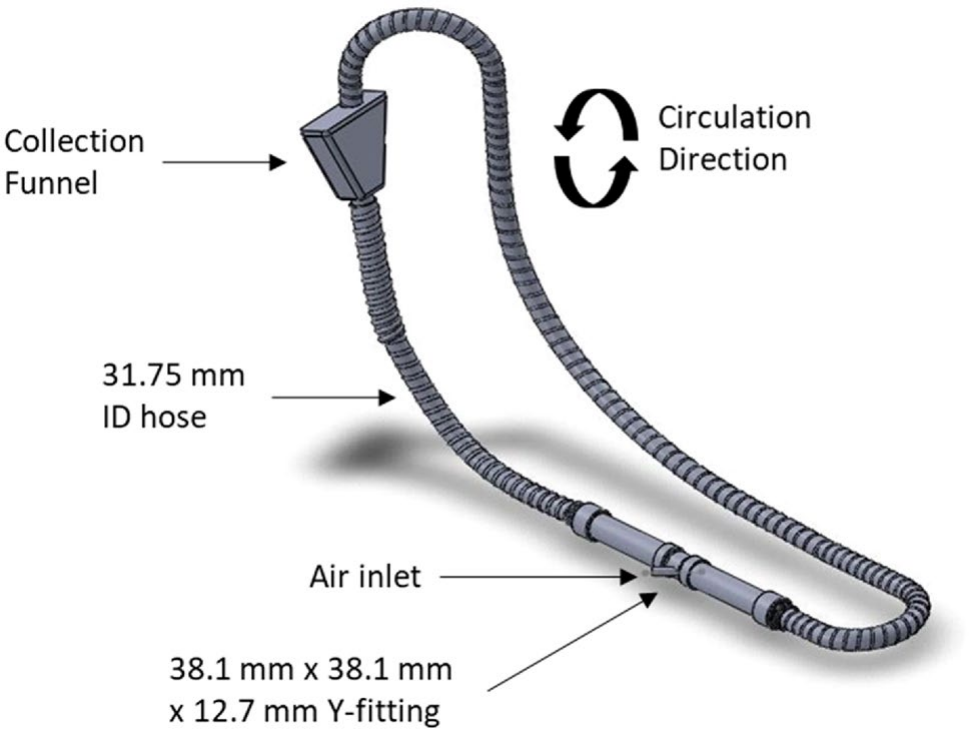
Computer aided design (CAD) of pneumatic recirculation system used to assess granule robustness.

To determine a suitable recirculation method for the Casoron® G4 granules, a series of tests were carried out to assess product degradation. As the pneumatic product delivery system was already inherent to the applicator, this was the preferred option for recycling the product as it would reduce system complication and upgrade costs. A small-scale pneumatic system was developed to recirculate granules continuously to assess their degradation potential. The system (Fig. 1) consisted of 4.87 m of 31.75 mm inner diameter (ID) spiral flex hose, a 38.1 mm × 38.1 mm × 12.7 mm ABS Y-fitting and a 360 mm × 150 mm × 430 mm collection funnel. Compressed air was introduced at the Y-fitting from a booster tank which fluctuated from 5.38 to 8.20 bar throughout the experiment. The booster tank was charged by a DeVilbiss 445 series compressor pump (DV Systems, Barrie, Canada). The air speed observed during the experiment ranged from 29 to 44 m s^-1^. This air speed is approximately 3.5 to 5 times greater than what is achievable with the applicator and was chosen to assess an extreme scenario and provide a considerable safety factor.

Three different mediums were selected for analysis, Casoron® G4 (MacDermid Agricultural Solutions Inc., Waterbury, CT, USA), 9–30-11 NPK MESZ fertilizer, and clay filler. Casoron® G4 is comprised of 50–70% silicon dioxide, 10–20% aluminium dioxide, 4% dichlobenil, 1–5% diiron trioxide, 1–5% magnesium oxide, 1–5% calcium oxide, 0.1–1% kaolin and 0.1–1% titanium dioxide. The fertilizer is comprised of 9% nitrogen, 30% phosphate, 11% potash, 0.4% magnesium, 7.9% sulfur, 0.8% zinc and 40.9% clay filler and other trace minerals and micronutrients^16^. The clay filler is comprised of < 3% copper sulfate, < 0.7% zinc sulfate and < 0.3% zinc with the rest being the clay itself^17^. The 9–30-11 NPZ MESZ fertilizer is a standard formulation used in wild blueberry production and was therefore included to assess its potential to be used with the designed applicator discussed later in the methodology. The clay filler was included to act as a control product given its relatively uniform nature and common use as a filler product in many granular fertilizers and pesticides.

One litre of granules were measured out and the bulk density was analyzed from nine 100 mL samples. 100 mL samples were weighed using a Denver Instruments TP-1502 (Sartorius AG, Gottingen, Germany) analytical balance. The granules were then introduced to the cycling apparatus and cycled pneumatically for 1 hour. Following cycling, the granules were emptied from the recirculation system and once again, nine 100 mL samples were analyzed according to their bulk density. This was repeated 3 times for each granule. The basis of the analysis is that, if granules were degrading, the bulk densities would be significantly greater following cycling. If the Casoron® G4 granules were degrading significantly, then an alternative conveyance method would be needed in the applicator design. In addition to the bulk density, 25 randomly selected granules were measured between their two furthest vertices both before and after the cycling period. For both measurements, results were compared with a two-sample t-test on whether the difference between means was less than zero, with significance determined at α = 0.05.

### Applicator considerations

The basis for the design of the spot applicator was a Valmar 1255 Twin Roller. Specifications for the stock configuration of this applicator are shown in Table 1.

**Table 1:** Specifications for stock Valmar 1255 Twin Roller.

Component	Description
Material	Stainless steel
Hopper	0.51 m^3^
Boom Width	7.31 m
Boom Height	Adjustable (0.56–0.86 m)
Boom Type	Self-levelling
Boom Orifices	12
Hosing	31.75 mm ID spiral flex
Fan Impeller	0.46 m
Impeller rpm	3,200–4,000 rpm
Impeller Drive	Honda GX 270 (6.3 kW at 3600 rpm net power)
Metering	Ground driven
Metering Drive	Hydraulic motor
Sections	2 metering (left boom, right boom)
Rollers	12 and 28 groove options
Height	1.82 m
Width (closed)	2.08 m
Length	2.61 m
Weight	489.88 kg

The applicator is configured to convey product pneumatically using a combination of a venturi, a rear mounted blower fan, and a Honda GX 270 gas engine (Honda Motor Co., Ltd., Tokyo, Japan). The first decision point in the design of the applicator was to determine whether this conveyance system could be used as part of a recirculation system. However, this is only feasible if the product can sustain the forces associated with recycling the product through the system. If the product breaks down during recycling, then the application rate will change independent of any changes to the system. A trial was therefore set up to test Casoron® G4 and its resistance to degradation.

### Recirculation system

The stock configuration of the applicator utilised 31.75 mm ID spiral flex hosing to convey product from the venturi to each of the 12 boom orifices. To assess the potential for product recirculation using the stock blower fan, additional hosing was connected at the boom orifices to return product to the hopper. The length of additional hosing varied from 2.50 to 5.50 m dependant on the distance the orifice was from the hopper. The stock configuration uses a 1:1 gearing ratio between the motor and blower fan. In initial testing, there was some product buildup in the return lines as the product made its way back to the hopper. For this reason, the gearing ratio was adjusted to 1.2:1. This moved the peak rpm of the blower fan from 3600 to 4320. After the adjustment there was no observed buildup in the system.

### Valve system

**Figure 2:**
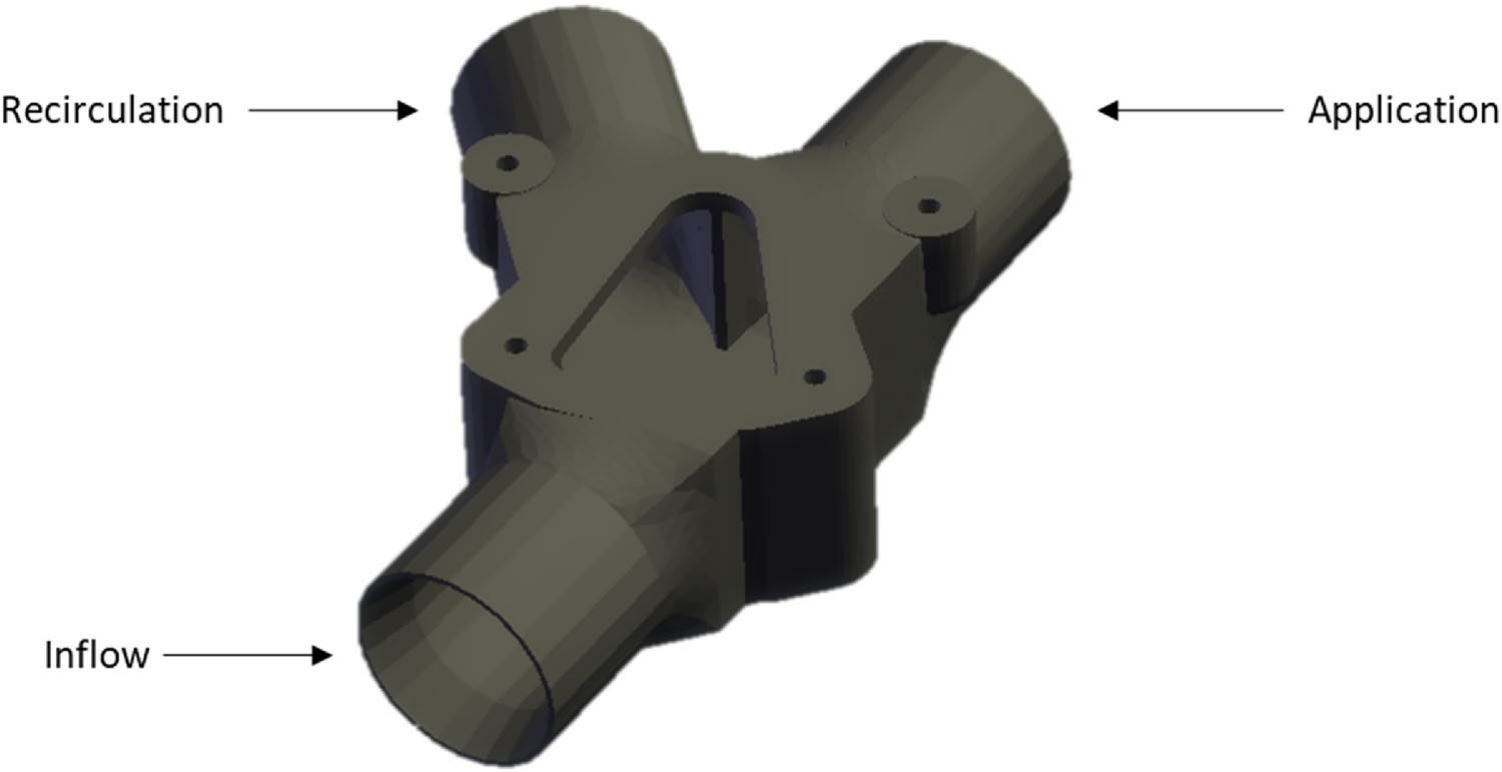
Computer aided design (CAD) drawing of the custom valves with the top retaining cover and internal gate removed.

Custom valves were designed as a means of directing product flow either onto the deflector plates or back through the recirculation hoses (Fig. 2).

The inflow and application ports are in line with one another to have as little effect on product flow as possible. As deflector plates are used in the design, it is imperative that the velocity of the product within the hoses is maintained to ensure proper product fanning. The recirculation hose is set at a 42° angle from the inline ports. Each of the ports has an outer diameter of 31.75 mm and hoses were stretched slightly so that the ports sat inside the hoses. All hoses were affixed to the valves with hose clamps. The inner diameter of the ports is 25 mm but tapers to 29.25 mm at the port edges to have minimal impact on product flow. A metal gate affixed to a Geeplus BRS50C39-6 bistable rotary solenoid (Geeplus, Beckenham, United Kingdom, BRS) was placed inside the central chamber of the valve. The BRSs operate at 24 V and rotate the gate 30° between two positions within the chamber. The switch between positions occurs in less than 10 ms. The two gate positions exclude product flow from either the recirculation or application port depending on the input signal.

### Control system

#### Signal input system

The control system was designed in three parts. The first utilised a John Deere (JD) RC2000 rate controller, Starfire 6000 receiver, SF3 correction signal, Gen-4 display and swath control (John Deere, Moline, United States). RC2000 settings can be observed in Appendix 1. This set of hardware was selected so that prescription maps could be read, and signals could be sent to each of the 12 boom orifices based on their georeferenced position. Georeferencing was done using a combination of the Starfire 6000 receiver and the SF3 correction signal. This system has previously demonstrated accuracies of ± 73.8 mm in wild blueberry fields^18^. Given the novelty of the developed applicator, the RC2000 does not support spot applicators. For this reason, the rate controller was configured as a liquid constant flow, self propelled sprayer. It should be noted that the georeferencing of the orifice positions does not use this denomination, rather, orifice locations were set up as a tow-behind sprayer so that the theoretical orifice locations would adjust accordingly while cornering. This was done in the display software and not the rate controller software. The use of the liquid constant flow setting was essential as it allowed for several pressure and flow gauges to be bypassed in the software. In alternative configurations, the JD software considers these essential checks and will shut off the system if the checks are not within a defined range. Swath control is likewise a vital component of the design as sectional control is not possible without it. Prescription maps were developed using the methodology described in^19^. Using the maps, the system sends up to 12 (one for each boom section), 12 V signals when the georeferenced orifice locations enter a management zone where product is required.

#### Signal processing and control system

The second component of the control system receives signals from the input system, processes them, and sends a second signal to actuate the orifices. This is performed based on the state change of the incoming signals from either 0 to 12 V or from 12 to 0 V. Six Arduino UNO R3 microcontrollers (one for every two boom sections) check for this state change and in combination with six Pololu Dual G2 High-Power Motor Drivers (Pololu Corporation, Las Vegas, United States) control the sending of either a + 24 or − 24 V pulse to the BRSs. Pulse lengths of 50 ms were used as this is still five times longer than the rated actuation time and mitigates overheating of the solenoid coils. The 24 V power was provided by an SEC America Model 695CE (SEC America Corporation, South Burlington, United States) high powered 12–24 V DC-DC boost convertor connected directly with the tractor’s battery. Control logic for the valves can be seen in Table 2.

**Table 2:** Control logic governing valve actuation.

Input signal voltage change	Voltage to solenoid	Orifice position
0 to 12 V	+ 24 V	Application
12 to 0 V	− 24 V	Recirculation

#### Rate control system

Product application rate was controlled by an Accu-Rate Controller Model 307,583 (Rawson Control Systems Incorporated, Oelwein, United States). Based on the entered parameters, the system controls a hydraulic metering device which, in combination with a wheel speed sensor, maintains the application at the desired rate. In all tests involving Casoron® G4, the system was run at 175 kg ha^-1^ to match the maximum application rate for wild blueberries^20^. The system was calibrated using the controller’s built in calibrate function which runs the metering device for a defined distance based on a fixed ground speed. Granules were collected from each of the orifices and weighed. Based on the measured weight and theoretical application area, the system was adjusted to ensure all orifices were applying the correct amount of product.

#### System interfacing

**Figure 3:**
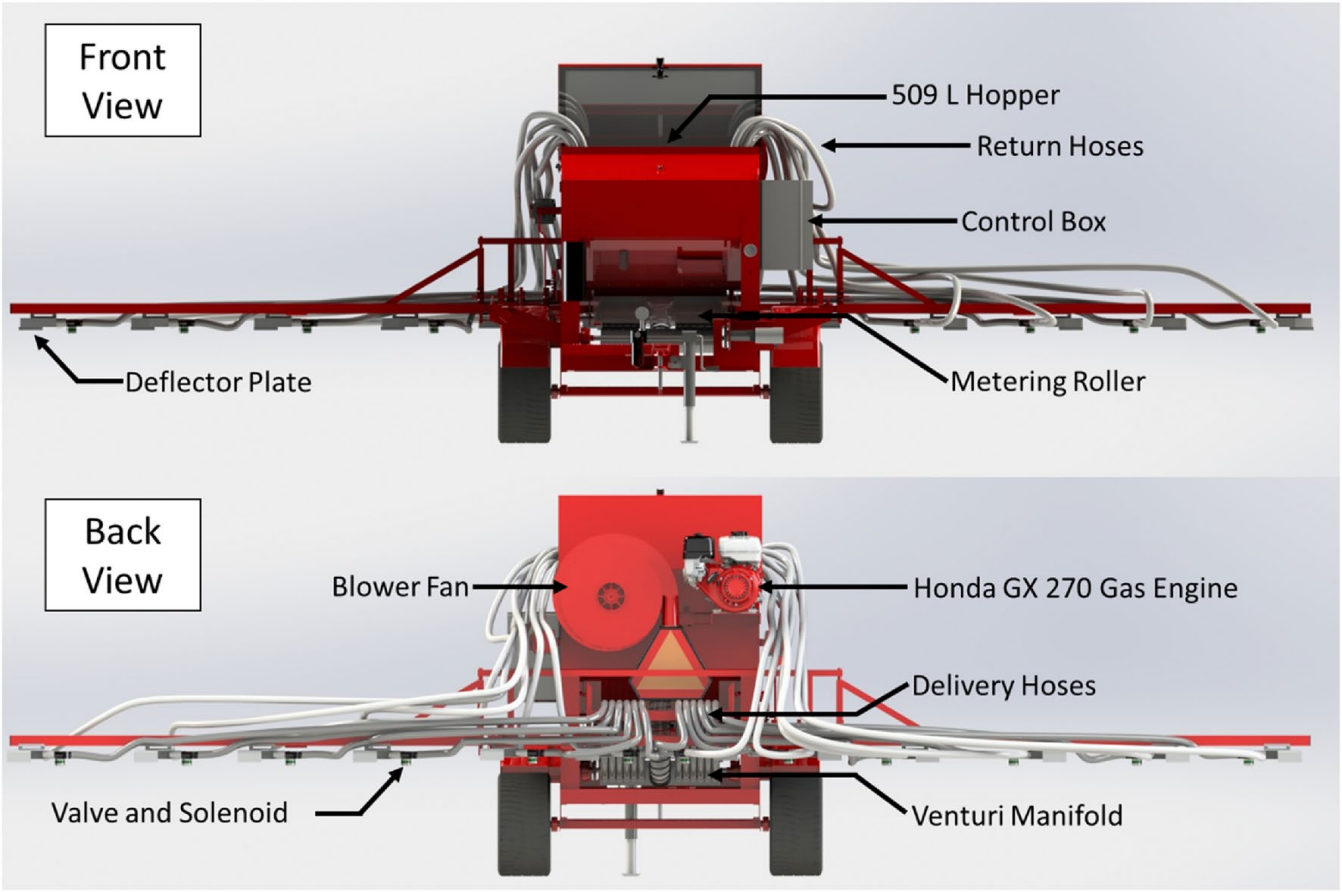
Computer aided design (CAD) drawing of the developed applicator showing both a front (top) and back (bottom) view.

**Figure 4:**
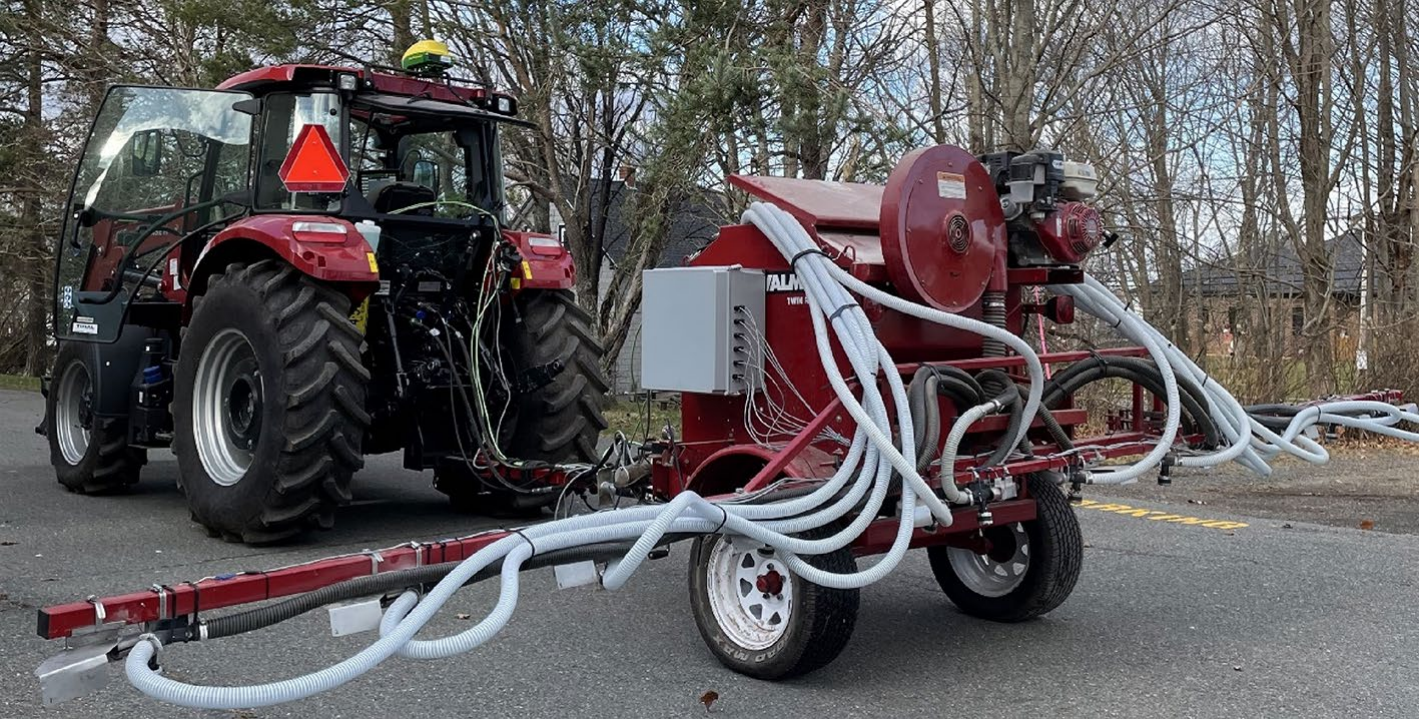
Prototype applicator during lab evaluation.

With each of the systems functioning in isolation, interfacing the systems was the final stage of development. The DC-DC boost converter, RC2000 controller, JD display and Accu-Rate controller were all mounted in the cabin of a Case IH Vestrum 130 (Case IH, Felton, United States). The Starfire 6000 was mounted on the roof of the tractor and orifice positions were georeferenced to its mounted location. All wiring was run out the back window port of the tractor, along the tow bar and into a weathertight control box mounted on the side of the applicator. All the circuitry controlling valve actuation was located inside the control box. Twelve signal wires were run out the back of the control box to provide control voltage for the solenoids. A CAD drawing of the applicator can be seen in Fig. 3 and a full image of the prototype applicator in Fig. 4.

### Lab evaluation of applicator

The developed applicator was lab evaluated to ensure product could be recycled without any blockages occurring within the system. To do this, the valves were left on recirculation and the system was run for 1 hour. The total number of blockages were recorded, and the experiment was repeated five times. A similar approach was used to evaluate the valves. Product was cycled for one hour and valves were manually switched every minute. The total number of blockages and failures of the valves were recorded over the duration of the trial.

**Figure 5:**
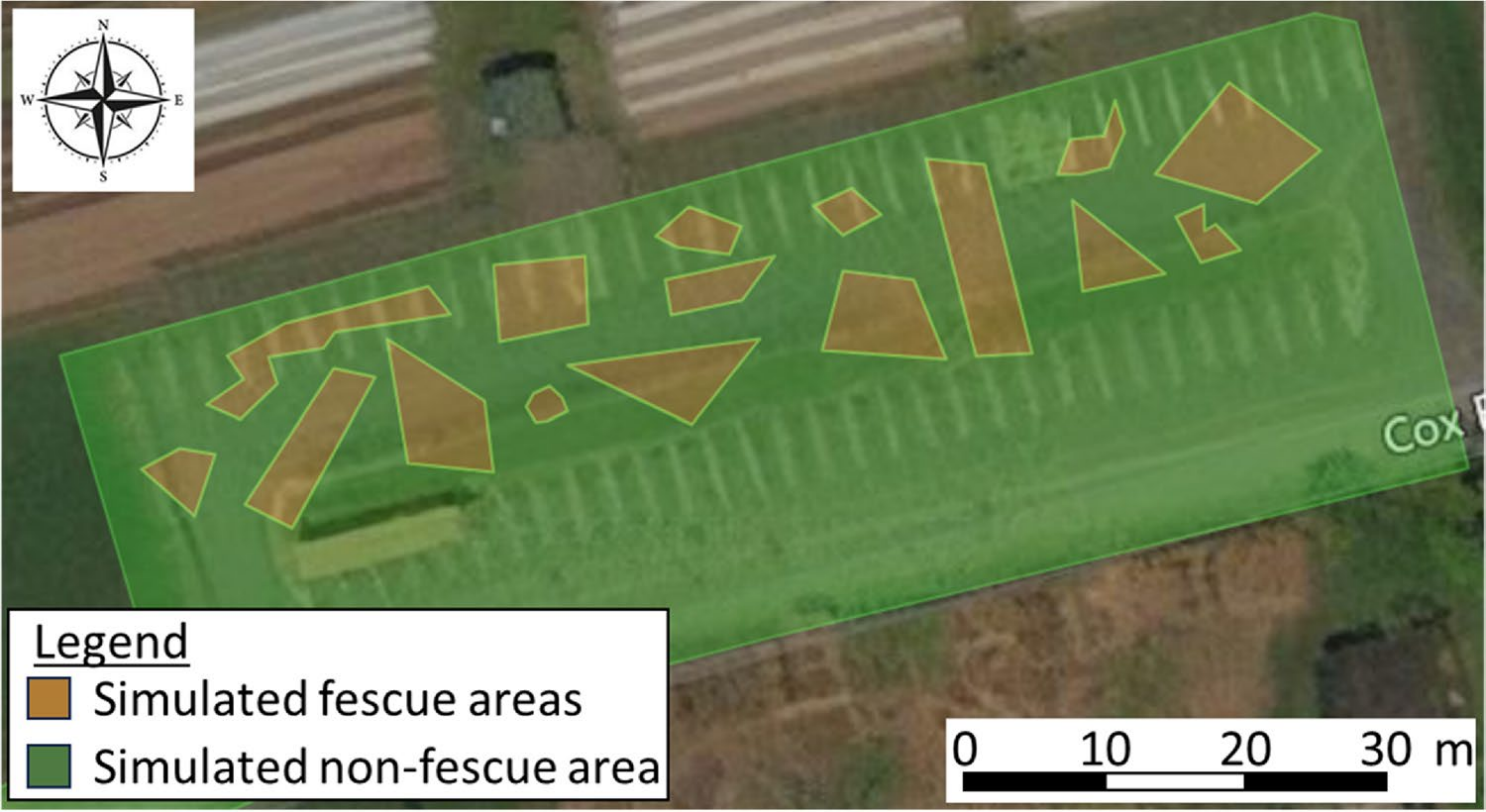
Prescription map of parking lot at Dalhousie Agricultural Campus, Bible Hill, Nova Scotia, Canada with arbitrarily drawn polygons for lab evaluating the applicators control and actuation systems. The green polygon simulates the non-fescue areas and the orange polygons simulate the fescue areas.

Valve actuation was further evaluated using a prescription map of a testing site at Dalhousie Agricultural Campus in Bible Hill, Nova Scotia (Fig. 5). Arbitrary polygons were drawn within the testing site boundaries to represent hair fescue.

A 50 m swath was defined within the testing site and the track was driven. At 10 randomly selected distances within the swath, all valves were checked to see if they were in the correct position based on the polygons from the prescription map. This trial was repeated 5 times with unique distances selected in each replicate. Throughout the trial, no product was applied, though the blower fan and all other components of the system were switched on.

A trial to assess application rate was likewise performed by randomly placing 10 Catchmaster glue traps (AP&G Co., Inc., Bayonne, USA) within a 50 m swath. The swaths were then driven, and product was applied uniformly. This was repeated across three swaths. Glue traps were analyzed using ImageJ 1.46^21^ in order to count the collected granules. These counts were then correlated to application rates from recounted ground truthed data.

## Results and discussion

### Granular degradation trial

Casoron® G4 bulk density was not altered during cycling (Table 3) and granule size was similar before and after cycling (Casoron® G4, the fertilizer and the clay filler each incurred an average decrease in granule length of 2.1%, 10.5%, and 19.4% respectively. Results from the fertilizer granule measurement, however, may be somewhat misleading, as visual observation of the post-cycling granules determined that the potash granules within the mixture were almost entirely broken apart, leaving only a fine dust. This meant that these particles could not be measured accurately and were therefore omitted from the post-cycling analysis. Inclusion of these particles would likely lead to the conclusion that significant product breakdown did occur.

**Table 3:** Results of the cycling analysis showing the mean bulk density before and after cycling as well as the p-value denoting the significance of the difference between values (two-sample t-test).

Granule	Sampling time	Mean bulk density (g L^-1^)	Significance of difference
Casoron® G4	Before cycling	659.7	*p* = 0.110
	After cycling	663.4	
Fertilizer	Before cycling	1015.8	*p* < 0.001
	After cycling	1064.5	
Clay Filler	Before cycling	1548.9	*p* < 0.001
	After cycling	1682.3	

Table 4). Fertilizer and clay filler, however, had greater bulk density and smaller granule length after cycling (Table 3 and Casoron® G4, the fertilizer and the clay filler each incurred an average decrease in granule length of 2.1%, 10.5%, and 19.4% respectively. Results from the fertilizer granule measurement, however, may be somewhat misleading, as visual observation of the post-cycling granules determined that the potash granules within the mixture were almost entirely broken apart, leaving only a fine dust. This meant that these particles could not be measured accurately and were therefore omitted from the post-cycling analysis. Inclusion of these particles would likely lead to the conclusion that significant product breakdown did occur.

**Table 4:** Results of the cycling analysis showing the mean granule length (measure between the two furthest vertices) before and after cycling as well as the p-value denoting the significance of the difference between values (two-sample t-test).

Granule	Sampling time	Granule length (mm)	Significance of difference
Casoron® G4	Before cycling	0.98	*p* = 0.378
	After cycling	0.96	
Fertilizer	Before cycling	4.32	*p* = 0.086
	After cycling	3.91	
Clay Filler	Before cycling	4.44	*p* = 0.003
	After cycling	3.72	

Table 4), suggesting these products degraded because of physical stresses incurred during cycling. That said, the change in fertilizer granule length was only marginally significant (Casoron® G4, the fertilizer and the clay filler each incurred an average decrease in granule length of 2.1%, 10.5%, and 19.4% respectively. Results from the fertilizer granule measurement, however, may be somewhat misleading, as visual observation of the post-cycling granules determined that the potash granules within the mixture were almost entirely broken apart, leaving only a fine dust. This meant that these particles could not be measured accurately and were therefore omitted from the post-cycling analysis. Inclusion of these particles would likely lead to the conclusion that significant product breakdown did occur.

Table 4). Casoron® G4, the fertilizer and the clay filler each incurred an average increase in bulk density of 0.5%, 4.8%, and 8.6% respectively.

Casoron® G4, the fertilizer and the clay filler each incurred an average decrease in granule length of 2.1%, 10.5%, and 19.4% respectively. Results from the fertilizer granule measurement, however, may be somewhat misleading, as visual observation of the post-cycling granules determined that the potash granules within the mixture were almost entirely broken apart, leaving only a fine dust. This meant that these particles could not be measured accurately and were therefore omitted from the post-cycling analysis. Inclusion of these particles would likely lead to the conclusion that significant product breakdown did occur.

In all, the cycling analysis confirmed Casoron® G4’s potential to be cycled pneumatically without significant product breakdown. This result justified the resulting development of the applicator using a pneumatic recycling system. The hope that the system could likewise serve as an applicator for fertilizer seems unlikely given the brittle nature of the mixture, in particular the potash granules. Alternative fertilizer brands or mixtures may help to mitigate this issue and allow the applicator to apply fertilizer spot specifically.

### Lab evaluation of applicator

With the valves left in the recycle position and product cycled for one hour, there were zero observed instances where product built up or back flowed within the delivery or return lines. Similarly, when testing the valves, there were zero observed instances where the valves jammed or failed to open/close when toggled on the JD display. Previous iterations of the design encountered difficulties in both areas so success in these trials was deemed sufficient as a final design. Those previous tests involved longer return hoses, a reduced pulley ratio on the blower fan, valves with longer chambers/gates, and valves which allowed for product to buildup behind the gate’s mounting point. With these issues resolved, the final design performed perfectly in isolation.

In testing the prescription maps and the ability for the system to turn on/off the correct orifices, there were zero instances where the system failed to operate as intended. This result demonstrated the potential of the mapping approach and the ability for the system to be highly precise and accurate. Given the success of the design, future work should look at in-field evaluations of the system at full scale.

Assessing the application rate of the applicator considered a target application rate of 175 kg ha^-1^. In testing, the mean application rate achieved was 182 ± 8.31 kg ha-1, which was not significantly different from the target rate (*p* = 0.415). This demonstrated that the system could still deliver the target application rate for Casoron® G4 independent of the alterations made to the system.

Other implementations should be explored to expand the applicator’s potential use cases, such as alternate cropping systems or agrochemicals. Cranberry is the evident option, given the similarities to wild blueberries and the documented use of dichlobenil^22–26^. That said, other cropping systems may have use cases for dichlobenil which are not currently employed due to the herbicide’s cost. With the development of the novel spot applicator, this may create opportunities for future dichlobenil implementation. In addition to dichlobenil, the developed applicator could be compatible with other granular herbicides like trifluralin (Bonanza® 10G, Rival 10G, Treflan® TR-10), a Group 3 herbicide effective against grasses and broadleaf weeds in various field crops^27^. Similarly, the technology might be adapted for granular insecticides such as tefluthrin (Force® 6.5G), bifenthrin (Bifen L/P), and chloropyrifos (Saurus®), provided suitable applications are determined. Further research is necessary to explore these potential uses. Given that these products are pesticide infused granules, similar to Casoron® G4, it is anticipated that their degradation would follow a similar profile to Casoron® G4. This would need to be confirmed before implementation.

## Conclusions

In analyzing the robustness of Casoron® G4 granules there was no significant product degradation when cycled pneumatically for one hour. This result was confirmed using both bulk density and by measuring particle size. This result supports the use of pneumatic conveyance as a means of recycling product for accommodating spot application with the precision applicator.

In testing the prescription maps and the ability for the system to turn on/off the correct nozzles, there were zero instances where the system failed to operate as intended. This result demonstrated the potential of the mapping approach and the ability for the system to be highly precise and accurate. Given the success of the design, future work should look at in-field evaluations of the system at full scale.

### Supplementary Information

Supplementary Information.

## Data Availability

The datasets used and/or analysed during the current study are available from the corresponding author on reasonable request.
