# Supplementary material for: Development of a novel precision applicator for spot treatment of granular agrochemical in wild blueberry
Source: Sci Rep. 2024 Jun 14;14:13751. doi: 10.1038/s41598-024-64650-z (PMC11178806; doi:10.1038/s41598-024-64650-z)
Supplement: Supplementary file 1 — Supplementary Information. [file 41598_2024_64650_MOESM1_ESM.docx]

# Appendix

Appendix 1: RC2000 settings used in the design and evaluation of the precision spot applicator

| RC2000 Settings | |
| --- | --- |
| Application Width | 24 ft |
| Machine Type | Self Propelled Sprayer |
| Application Mode | Liquid Constant Flow |
| Number of Sections | 12 |
| Section Valve Type | 3-wire |
| Equal Width Sections | yes |
| Fence Rows Enabled | no |
| Section Width | 2 ft |
| Pressure Sensor | no |
| Agitator Valve | no |
| Flow Return Installed | no |
| Control Valve Type | none |
| Flowmeter Calibration | 8000 |
| Flowmeter Pulse Units | 10 gal |
| Tank Capacity | 0 |
| Current Level | 0 |
| Low Tank Level | 0 |
| Tank Fill Monitor | 0 |
| Preset Rate Value | 175 |
| Rate Bump | 0 |
| Rate Selection | Map Based |
| Rate Smoothing | 15% |
| Decimal Shift | 1 |
| Off Rate Alarm | 30 |
| Alarm? | no |
| Minimum Flow Rate | 0 |
